# Effects of early vs. late administration of labetalol on maternal cerebral perfusion and fetal growth in severe pre-eclampsia

**DOI:** 10.3389/fmed.2026.1728192

**Published:** 2026-03-02

**Authors:** Jiahui Wang, Fangfang Yu

**Affiliations:** Obstetrics and Gynecology Department, The First Affiliated Hospital of Soochow University, Suzhou, Jiangsu, China

**Keywords:** antihypertensive therapy, cerebral perfusion, labetalol, maternal outcomes, neonatal outcomes, severe pre-eclampsia

## Abstract

**Background:**

Severe pre-eclampsia is a major contributor to maternal and perinatal morbidity and mortality worldwide. Antihypertensive therapy is essential, yet the impact of the timing of intravenous labetalol initiation on maternal cerebral hemodynamics and clinical outcomes remains uncertain, particularly in Chinese populations.

**Methods:**

In this retrospective cohort study, we evaluated women with severe pre-eclampsia treated at the Department of Obstetrics and Gynecology, The First Affiliated Hospital of Soochow University, Jiangsu Province, China, between March 2023 and July 2025. Women diagnosed with severe pre-eclampsia were categorized into two groups based on the timing of labetalol administration: early (≤60 min after diagnosis) and late (>60 min). Clinical characteristics, laboratory data, treatment details, and outcomes were retrieved from electronic records. Primary outcomes were changes in middle cerebral artery pulsatility index (MCA PI) and time to blood pressure control. Secondary outcomes included maternal complications (HELLP syndrome, acute kidney injury, pulmonary edema, intensive care unit admission, and maternal death) and neonatal outcomes (birthweight, small-for-gestational-age, NICU admission, neonatal complications, and perinatal death). Statistical analyses employed Welch’s *t*-test, Mann–Whitney U test, *χ*^2^ or Fisher’s exact test, and correlation testing, with *p* < 0.05 considered significant.

**Results:**

Of 372 women screened, 320 were included (162 early-treatment and 158 late-treatment). Baseline demographic and laboratory profiles were comparable. Early labetalol initiation was associated with significantly greater increases in MCA PI at 60 min, 6 h, and 24 h (*p* ≤ 0.040) and with shorter median time to blood pressure control (60 vs. 95 min, *p* < 0.001). At 6 and 24 h, systolic and diastolic pressures were lower in the early group (*p* < 0.01). Improvement in MCA PI was inversely correlated with time to blood pressure control, suggesting faster hemodynamic stabilization with earlier treatment. Maternal complications and neonatal outcomes did not differ significantly between groups.

**Conclusion:**

Early initiation of intravenous labetalol was associated with improved maternal cerebral perfusion and more rapid blood pressure stabilization, without an increase in adverse maternal or neonatal outcomes. These findings highlight the importance of timely antihypertensive therapy in severe pre-eclampsia, although prospective studies are required to establish causality and long-term clinical benefit.

## Introduction

Hypertensive disorders of pregnancy (HDP) remain a leading cause of maternal and perinatal morbidity and mortality worldwide, accounting for a substantial proportion of preventable maternal deaths (1–3). Among these conditions, pre-eclampsia—defined as new-onset hypertension after 20 weeks of gestation accompanied by proteinuria or evidence of maternal organ dysfunction—affects approximately 2–8% of pregnancies globally and continues to exert a disproportionate burden on maternal and neonatal outcomes (4, 5). Severe pre-eclampsia is associated with life-threatening complications, such as eclampsia, intracerebral hemorrhage, pulmonary edema, acute kidney injury, placental abruption, and disseminated intravascular coagulation, while neonates are at increased risk of prematurity, fetal growth restriction, low birth weight, and perinatal mortality (6–8).

Prompt control of severe hypertension is a cornerstone of management in pre-eclampsia, with the primary aim of reducing maternal cerebrovascular risk while preserving uteroplacental perfusion (2, 9). International guidelines consistently recommend initiation of antihypertensive therapy when systolic blood pressure reaches ≥160 mmHg or diastolic pressure ≥110 mmHg, ideally within 30–60 min of diagnosis (2, 10, 11). Intravenous labetalol is widely used as a first-line agent owing to its rapid onset of action, combined *α*- and *β*-adrenergic blockade, and favorable maternal–fetal safety profile, and it is commonly preferred in acute management settings (12, 13). However, while antihypertensive agents have been extensively studied, the clinical and physiological consequences of delayed initiation of therapy remain poorly characterized.

Despite consensus regarding treatment thresholds, the optimal timing of antihypertensive initiation in severe pre-eclampsia remains insufficiently defined. In real-world clinical practice, delays in treatment initiation are common and may prolong maternal exposure to critically elevated blood pressure, thereby increasing the risk of stroke, eclampsia, and other end-organ complications (14, 15). Observational data suggest that even short delays in antihypertensive therapy may be associated with adverse maternal neurological outcomes (16, 17). Conversely, earlier initiation of labetalol may facilitate more rapid hemodynamic stabilization, shorten time to blood pressure control, and potentially mitigate downstream maternal and neonatal risks. However, existing evidence directly comparing early vs. delayed antihypertensive initiation remains limited, and prior studies have largely focused on short-term blood pressure reduction rather than clinically meaningful maternal or neonatal outcomes (1, 12).

Cerebral hemodynamic disturbance has emerged as a critical contributor to maternal morbidity in severe pre-eclampsia. Impairment of cerebrovascular autoregulation increases susceptibility to hypertensive encephalopathy, cerebral edema, and intracerebral hemorrhage (18, 19). The middle cerebral artery pulsatility index (MCA PI), measured non-invasively using Doppler ultrasonography, serves as a surrogate marker of cerebrovascular resistance and autoregulatory function (20, 21). Maternal cerebral hemodynamics assessed by MCA Doppler provide a noninvasive window into cerebral autoregulatory function and may serve as an early surrogate marker of neurological risk in severe pre-eclampsia. Alterations in MCA PI have been associated with adverse maternal neurological outcomes, supporting its clinical relevance in the assessment of cerebrovascular vulnerability in women with severe pre-eclampsia (22, 23).

Importantly, data evaluating whether the timing of antihypertensive therapy influences cerebral perfusion indices, such as MCA PI, are scarce. Moreover, neonatal outcomes under different antihypertensive timing strategies remain poorly characterized, with most studies underpowered to assess downstream perinatal effects (9, 24). These evidence gaps are particularly pronounced in Asian populations, despite known differences in genetics, clinical practice patterns, and healthcare systems (4, 21).

Accordingly, this study aimed to compare early vs. delayed intravenous labetalol administration in women with severe pre-eclampsia, focusing on maternal cerebral perfusion, blood pressure stabilization, and neonatal outcomes. By integrating cerebral hemodynamic parameters with clinically meaningful maternal and perinatal endpoints, this investigation seeks to address a critical knowledge gap regarding the physiological and clinical consequences of treatment timing. Using data from a large Chinese cohort, the study aims to provide robust, population-specific evidence to inform the optimal timing of antihypertensive therapy in severe pre-eclampsia.

## Methodology

### Study design and setting

This retrospective cohort study was conducted at the Obstetrics and Gynecology Department, the First Affiliated Hospital of Soochow University, Jiangsu Province, China, from March 2023 to July 2025. The institution serves as a major referral hub for hypertensive disorders of pregnancy, with approximately 7,400 deliveries annually, thereby providing a representative patient population for this investigation.

The study was designed and reported in accordance with the STROBE (Strengthening the Reporting of Observational Studies in Epidemiology) guidelines. The research adhered to the principles of the Declaration of Helsinki (2013 revision). Given the retrospective nature of the study, the requirement for individual written consent was waived.

### Participants

Women were eligible if they were diagnosed with severe pre-eclampsia after 20 weeks of gestation, defined according to the criteria of the American College of Obstetricians and Gynecologists (6) and the Chinese Society of Obstetrics and Gynecology. Severe pre-eclampsia was diagnosed in the presence of sustained systolic blood pressure ≥160 mmHg or diastolic blood pressure ≥110 mmHg on two occasions, at least 4 h apart, or evidence of maternal end-organ dysfunction such as thrombocytopenia, impaired hepatic or renal function, pulmonary edema, or new-onset neurological or visual symptoms.

- *Inclusion criteria*: Women aged ≥18 years with a diagnosis of severe pre-eclampsia after 20 weeks of gestation.
- *Exclusion criteria*: Multiple pregnancy; known major fetal anomalies; pre-existing cardiovascular, renal, or cerebrovascular disease; contraindications to *β*-blocker therapy (e.g., severe asthma or advanced atrioventricular block); incomplete or missing clinical data; or refusal of the institutional management protocol.

### Exposure and clinical management

The exposure of interest was the timing of intravenous labetalol administration following diagnosis. Patients were classified into an early-treatment group, in which labetalol was initiated within 60 min, and a late-treatment group, in which administration occurred after 60 min. The 60-min threshold was selected *a priori* based on international guideline recommendations that severe hypertension in pregnancy should be treated within 30–60 min of confirmation to reduce maternal cerebrovascular risk (6, 8).

Time zero was defined as the documented diagnosis of severe pre-eclampsia in the electronic medical record. Treatment timing was extracted from electronic medical records using time-stamped diagnosis and medication-administration data.

Clinical management was standardized across groups. Labetalol was administered as an initial bolus of 20–40 mg intravenously, repeated at 10–20 min intervals if blood pressure remained uncontrolled, followed by continuous infusion or repeated boluses at the discretion of the treating physician. Adjunctive antihypertensives (hydralazine, nifedipine, or nicardipine) were used when necessary. Magnesium sulfate was administered for seizure prophylaxis unless contraindicated, and antenatal corticosteroids were provided to women with gestational age below 34 weeks. Delivery timing, mode, and anesthesia type were determined according to maternal and fetal status.

### Data collection

Clinical data were extracted from electronic medical records by two independent investigators using a standardized form, with discrepancies resolved by consensus. Information collected included maternal demographics (age, body mass index, parity, gestational age at diagnosis, aspirin use, and comorbidities), clinical presentation (oliguria, pulmonary edema, and time of admission), and baseline laboratory values (platelet count, lactate dehydrogenase, aspartate aminotransferase, alanine aminotransferase, serum creatinine, uric acid, and urine protein-to-creatinine ratio).

Management and treatment details recorded were timing and dosage of labetalol, use of adjunctive antihypertensives, magnesium sulfate, and corticosteroids, as well as delivery mode and anesthesia type. Maternal outcomes included serial systolic and diastolic blood pressure, middle cerebral artery pulsatility index (MCA PI) at baseline, 60 min, 6 h, and 24 h, time to blood pressure control (defined as the interval from diagnosis to SBP < 160 mmHg and DBP < 110 mmHg), and maternal complications such as HELLP syndrome, acute kidney injury, pulmonary edema, ICU admission, maternal death, or adverse drug reactions. Neonatal outcomes included gestational age at delivery, birthweight, birthweight centile based on Chinese population standards, small-for-gestational-age status (<10th and <3rd centile), NICU admission, neonatal complications, and perinatal death.

Women with incomplete or missing essential clinical or laboratory records were excluded during the screening process (*n* = 52). For the final analytic cohort (*n* = 320), missing data for secondary variables (e.g., laboratory indices and adjunct therapy timing) were minimal (<5% for all variables). These cases were addressed using complete-case analysis. Sensitivity checks confirmed that exclusion of these cases did not materially alter the direction or significance of the study findings.

### Outcome measures

- Primary outcomes:
  - Change in MCA PI across serial assessments
  - Time to blood pressure control (interval from diagnosis to achieving SBP < 160 mmHg and DBP < 110 mmHg).
- Secondary outcomes:
  - Maternal: refractory hypertension, HELLP syndrome, acute kidney injury (KDIGO criteria), pulmonary edema, ICU admission, and maternal death
  - Neonatal: birthweight, small-for-gestational-age, NICU admission, neonatal complications, and perinatal death.

### Sample size estimation

The required sample size was estimated using the formula for comparing two independent means:


$$n=\frac{2\sigma^{2}{\left(Z_{1-\alpha /2}+Z_{1-\beta }\right)}^{2}}{\Delta^{2}}$$

where,

*σ* represents the assumed standard deviation of MCA PI change,

*Δ* the minimum clinically significant difference,

Z_1 − *α*/2_ is the critical value for a two-sided *α* = 0.05, and

Z_1 − *β*_ is the critical value for 80% power.

Assuming an SD of 0.15, a difference of 0.10, Z_1 − α/2_ = 1.96, and Z_1 − β_ = 0.84, the calculation yielded a requirement of 136 patients per group (272 total). The final cohort comprised 320 women (162 early-treatment and 158 late-treatment), exceeding this threshold and ensuring adequate statistical power.

### Statistical analysis

Continuous variables were assessed for normality using the Shapiro–Wilk test. Normally distributed variables were summarized as mean ± standard deviation and compared using Welch’s *t* test, whereas non-normally distributed variables were reported as median [interquartile range] and compared using the Mann–Whitney *U* test. Categorical variables were presented as counts and percentages and compared using the *χ*^2^ test or Fisher’s exact test.

Baseline balance was evaluated using standardized mean differences (≥0.20 considered meaningful). Relative risks with 95% confidence intervals (CI) were calculated for binary outcomes. Correlations between MCA PI and hemodynamic measures were assessed using Pearson or Spearman coefficients.

To determine the independent effect of early vs. late labetalol administration, multivariable logistic regression was performed for binary outcomes (maternal complications, neonatal adverse outcomes, and NICU admission) and multivariable linear regression for continuous outcomes (time to blood pressure control and MCA ΔPI at 60 min). Models were adjusted for maternal age, parity, gestational age, chronic hypertension, initial systolic blood pressure, magnesium sulfate use, and time from arrival to diagnosis. Covariates were selected *a priori* based on clinical relevance and prior literature. Adjusted effects are reported as aOR or *β* coefficients with 95% CIs.

All analyses were two-sided, with *p* < 0.05 considered significant. Statistical analyses were conducted using SPSS version 29.0 (IBM Corp.) and R version 4.3.2.

## Results

### Study population

**Figure 1:**
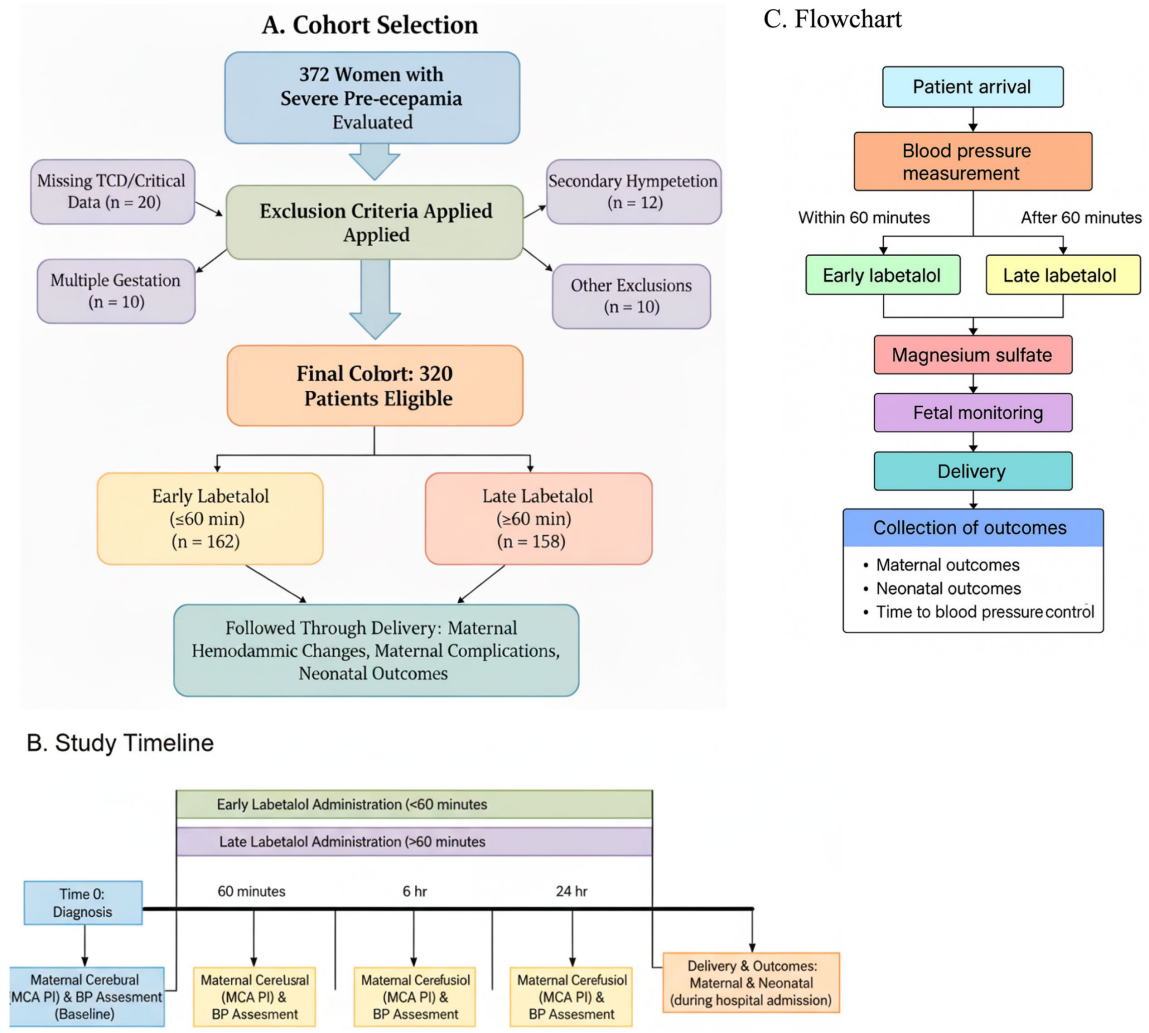
Cohort selection and study timeline. **(A)** Cohort selection showing screened patients (*n* = 372), exclusions for prespecified criteria (*n* = 52), and final analytic cohort divided into early labetalol (≤60 min, *n* = 162) and late labetalol (>60 min, *n* = 158). **(B)** Study timeline illustrating diagnosis of severe pre-eclampsia (time zero), early versus late labetalol initiation, and scheduled assessments of maternal cerebral perfusion (MCA PI) and blood pressure at 60 min, 6 h, and 24 h, with subsequent delivery and outcome evaluation. **(C)** Procedural flowchart. This panel illustrates the procedural pathway for women presenting with severe hypertension, such as initial assessment, timing of labetalol administration (≤60 min vs. >60 min), adjunct management, and subsequent outcome collection.

A total of 372 women with severe pre-eclampsia were screened, of whom 52 were excluded according to prespecified criteria. The final analytic cohort comprised 320 women, including 162 who received early labetalol (≤60 min after diagnosis) and 158 who received late labetalol (>60 min) (Figure 1). Baseline balance was assessed using standardized mean differences, with no clinically meaningful imbalance observed.

### Baseline demographics and clinical characteristics

**Figure 2:**
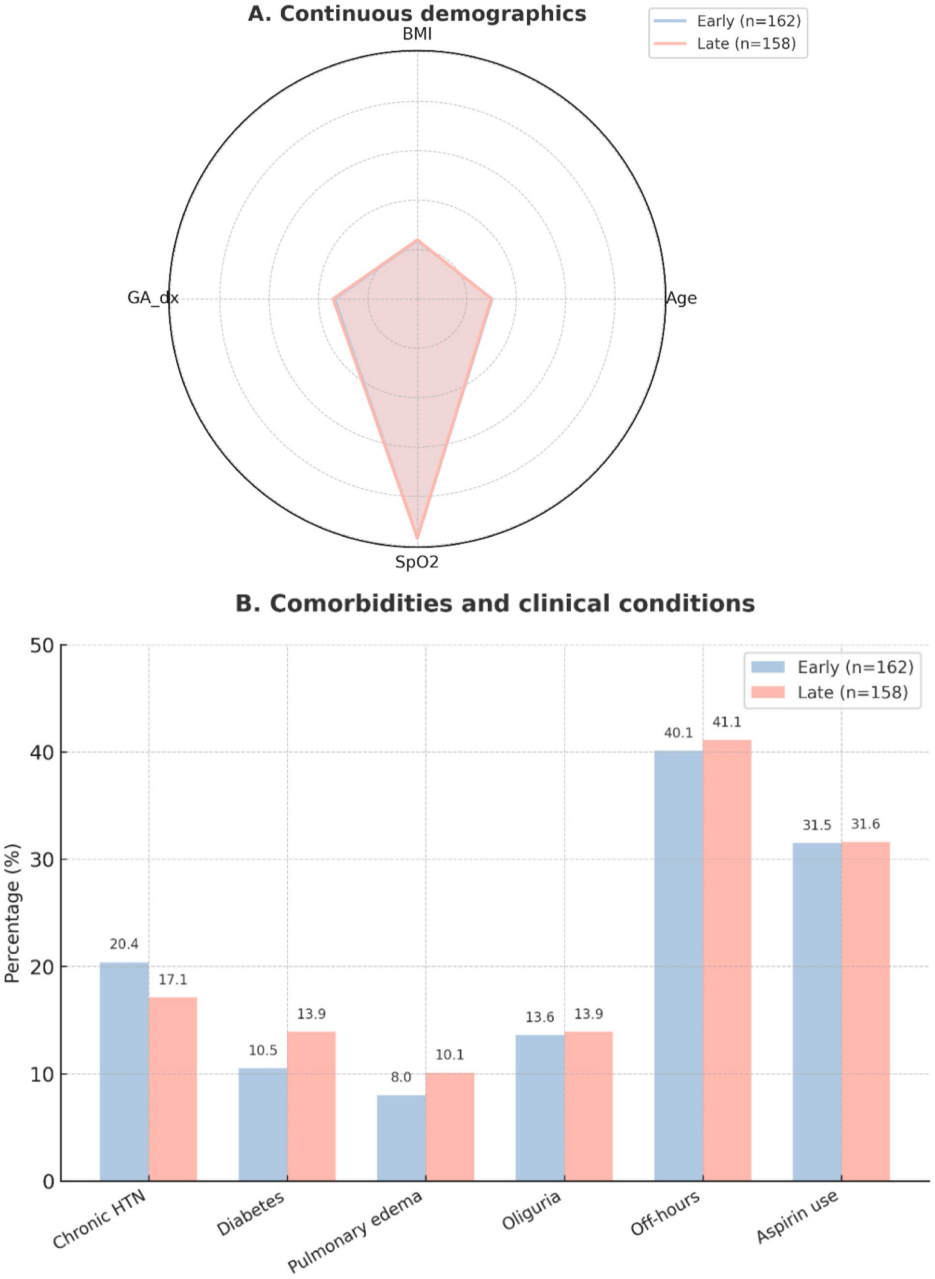
Baseline characteristics of study participants. **(A)** Distribution of continuous demographic and clinical variables, such as maternal age, pre-pregnancy BMI, gestational age at diagnosis of severe pre-eclampsia, and oxygen saturation (SpO_2_). Data are shown as mean ± SD; groups represent early labetalol (≤60 min, *n* = 162) and late labetalol (>60 min, *n* = 158). **(B)** Frequency of baseline comorbidities and clinical conditions, such as chronic hypertension, diabetes, pulmonary edema, oliguria, off-hours presentation, and aspirin use prior to admission. Values are expressed as percentages within each group.

Baseline maternal demographics, comorbidities, and clinical features were comparable between groups (Table 1). The mean maternal age was 30 years, and gestational age at diagnosis of severe pre-eclampsia was similar (33.5 vs. 34.1 weeks, *p* = 0.073). Rates of chronic hypertension, diabetes, oliguria, pulmonary edema, and off-hours presentation did not differ significantly. Approximately one-third of patients had used aspirin prior to admission. These findings are consistent with the distributions shown in Figure 2.

**Table 1:** Baseline demographics, clinical characteristics, and comorbidities.

Variable	Early (*n* = 162)	Late (*n* = 158)	*p*-value
Demographics			
Maternal age (years)	30.2 ± 4.5	29.9 ± 5.3	0.601
Pre-pregnancy BMI (kg/m^2^)	23.7 ± 3.4	23.9 ± 3.2	0.618
GA at severe PE diagnosis (weeks)	33.5 ± 3.0	34.1 ± 3.0	0.073
Clinical features			
SpO₂ (%)	96.9 ± 1.9	96.7 ± 1.9	0.230
Oliguria (<500 mL/24 h)	22 (13.6)	22 (13.9)	1.000
Off-hours presentation	65 (40.1)	65 (41.1)	0.943
Comorbidities			
Chronic hypertension	33 (20.4)	27 (17.1)	0.543
Diabetes (any)	17 (10.5)	22 (13.9)	0.443
Pulmonary edema	13 (8.0)	16 (10.1)	0.645
Aspirin use prior to admission	51 (31.5)	50 (31.6)	1.000

Values are mean ± SD or *n* (%). Continuous variables compared using Welch’s *t*-test; categorical variables compared using *χ*^2^ test or Fisher’s exact test when appropriate. A *p*-value <0.05 was considered statistically significant. GA, gestational age; BMI, body mass index; PE, pre-eclampsia; SpO₂, peripheral oxygen saturation.

### Baseline laboratory values

**Figure 3:**
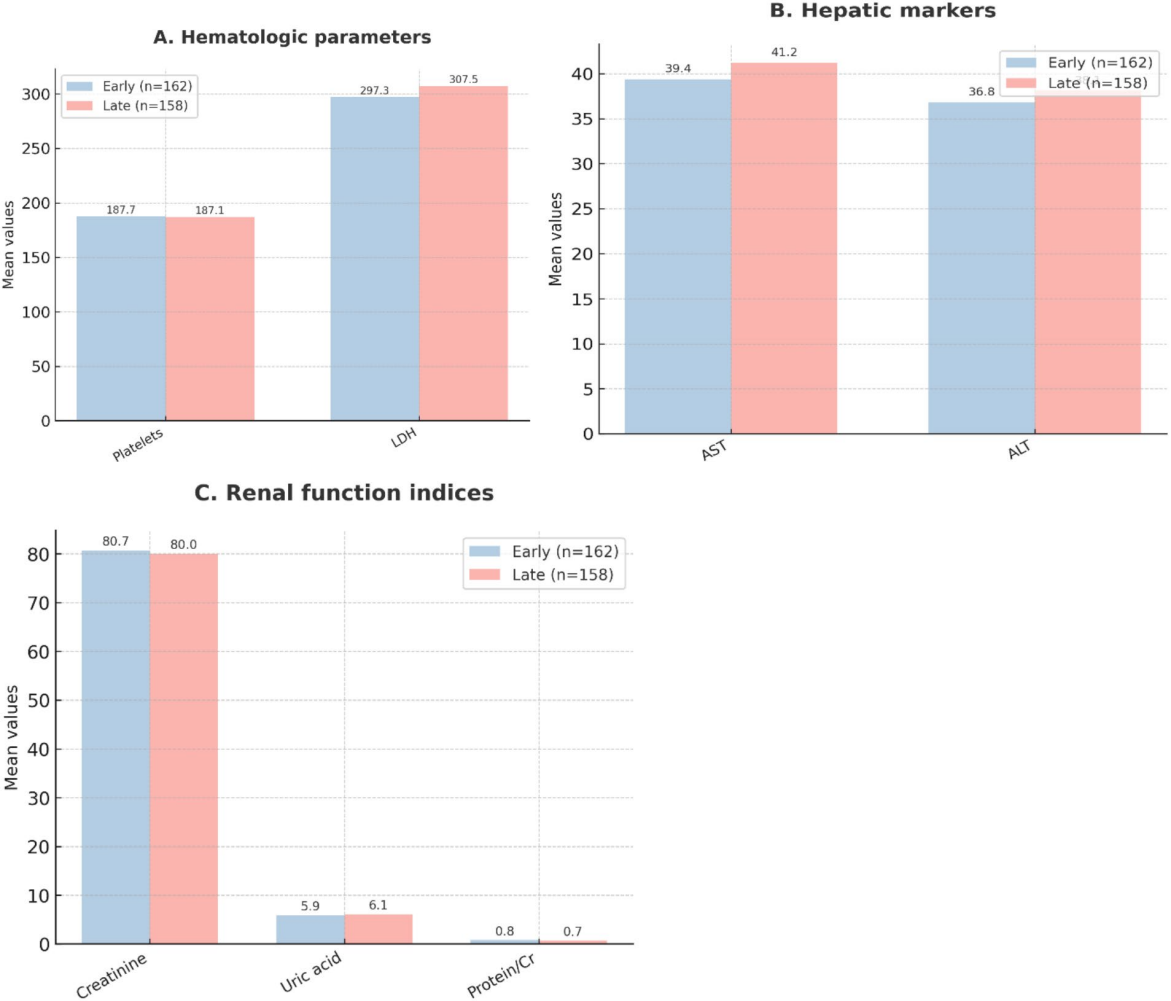
Baseline laboratory values. **(A)** Hematologic parameters, such as platelet count and lactate dehydrogenase (LDH). **(B)** Hepatic markers, such as aspartate aminotransferase (AST) and alanine aminotransferase (ALT). **(C)** Renal function indices, such as serum creatinine, uric acid, and urine protein-to-creatinine ratio. Values are presented as mean ± SD. Comparisons are between the early labetalol group (≤60 min, *n* = 162) and the late labetalol group (>60 min, *n* = 158).

Hematologic, hepatic, and renal laboratory parameters at presentation were also similar (Table 2). Platelet counts, liver enzymes (AST, ALT), serum creatinine, and uric acid levels did not differ significantly. The urine protein-to-creatinine ratio was slightly higher in the early-treatment group, though the difference was not statistically significant (*p* = 0.088). Figure 3 illustrates these laboratory distributions.

**Table 2:** Baseline laboratory values and severity markers.

Variable	Early (*n* = 162)	Late (*n* = 158)	*p*-value
Hematologic			
Platelets (10^9^/L)	187.7 ± 42.2	187.1 ± 45.8	0.914
LDH (U/L)	297.3 ± 98.9	307.5 ± 96.9	0.351
Liver function			
AST (U/L)	39.4 ± 18.2	41.2 ± 19.2	0.391
ALT (U/L)	36.8 ± 16.6	38.1 ± 17.8	0.489
Renal function			
Creatinine (μmol/L)	80.7 ± 24.7	80.0 ± 23.6	0.789
Uric acid (mg/dL)	5.9 ± 1.6	6.1 ± 1.5	0.190
Urine protein/creatinine ratio (mg/mg)	0.8 ± 0.5	0.7 ± 0.4	0.088

Values are mean ± SD. Continuous variables compared using Welch’s *t*-test. A *p*-value <0.05 was considered statistically significant. LDH, lactate dehydrogenase; AST, aspartate aminotransferase; ALT, alanine aminotransferase.

### Labetalol exposure and adjunctive management

Time to first labetalol dose was markedly shorter in the early group (33.8 vs. 108.9 min, *p* < 0.001; Table 3). Patients treated later required higher cumulative doses within the first 6 and 24 h (both *p* < 0.001). First-dose amounts were similar between groups.

**Table 3:** Labetalol exposure, management, and delivery.

Variable	Early (*n* = 162)	Late (*n* = 158)	*p*-value
Labetalol exposure			
Time to first dose (min)	33.8 ± 15.1	108.9 ± 42.9	<0.001*
First dose (mg)	53.5 ± 46.4	60.3 ± 56.5	0.243
Cumulative dose 0–6 h (mg)	112.5 ± 50.2	153.4 ± 72.5	<0.001*
Cumulative dose 0–24 h (mg)	195.5 ± 63.2	233.2 ± 85.7	<0.001*
Adjunct therapies			
Magnesium sulfate started (min)	10.9 ± 14.8	9.9 ± 16.1	0.609
Hydralazine	24 (14.8)	33 (20.9)	0.203
Nifedipine	31 (19.1)	39 (24.7)	0.287
Nicardipine	17 (10.5)	24 (15.2)	0.276
Magnesium sulfate given	113 (69.8)	116 (73.4)	0.547
Antenatal corticosteroids given	92 (56.8)	90 (57.0)	1.000
Expectant management	82 (50.6)	79 (50.0)	1.000
Delivery			
Vaginal	73 (45.1)	76 (48.1)	0.665
Cesarean	89 (54.9)	82 (51.9)	—
Anesthesia			0.048*
Spinal	75 (46.3)	57 (36.1)	
Epidural	53 (32.7)	67 (42.4)	
General	18 (11.1)	10 (6.3)	
None	16 (9.9)	24 (15.2)	

Values are mean ± SD or n (%). Continuous variables compared using Welch’s *t*-test; categorical variables compared using *χ*^2^ test or Fisher’s exact test when appropriate. *A *p*-value <0.05 was considered statistically significant. IV, intravenous. Cumulative dose refers to the total labetalol received within the stated time window after diagnosis of severe pre-eclampsia.

**Figure 4:**
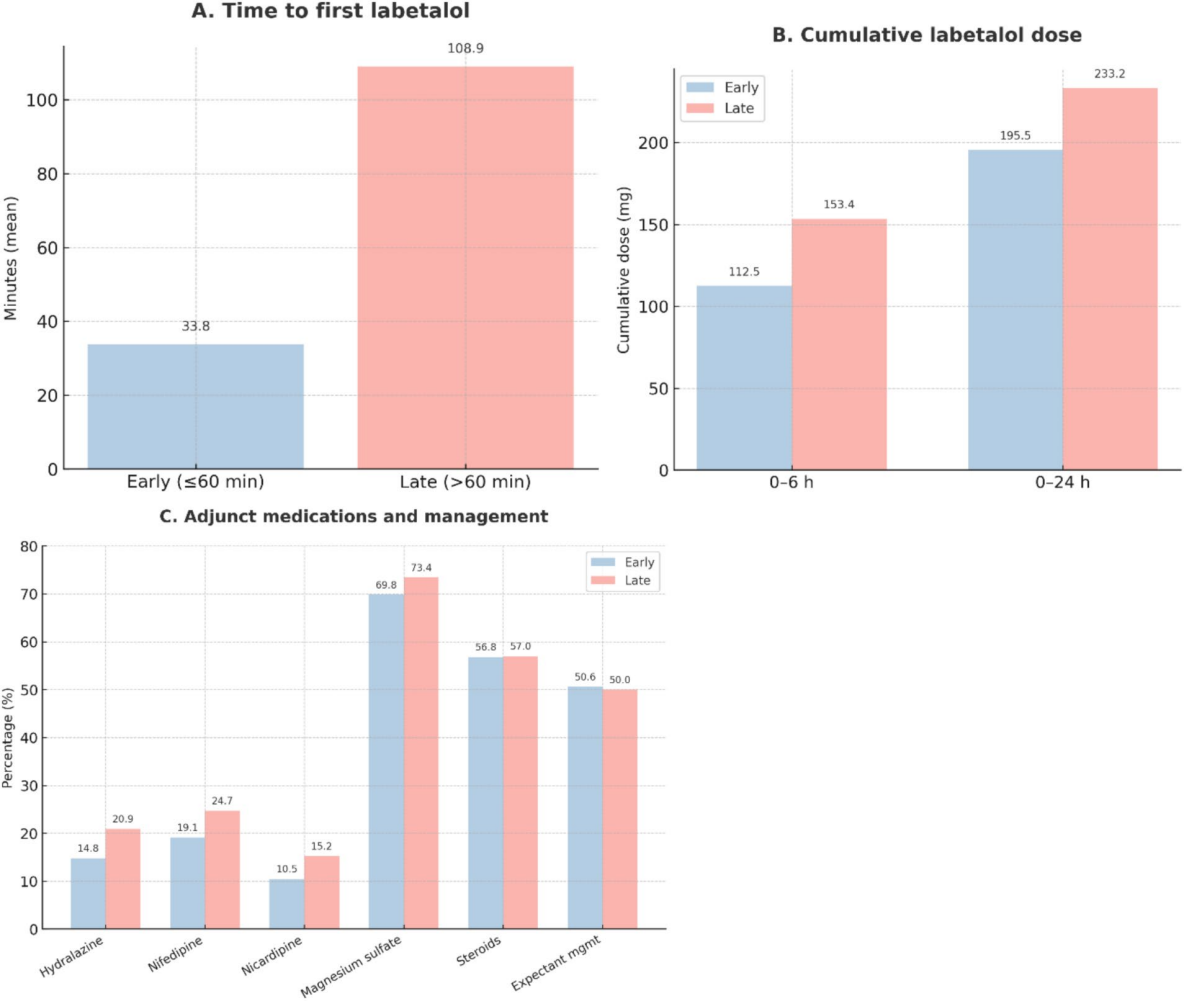
Labetalol exposure and adjunct management. **(A)** Distribution of time to first labetalol administration after diagnosis of severe pre-eclampsia, shown separately for early (≤60 min, *n* = 162) and late (>60 min, *n* = 158) groups.**(B)** Cumulative labetalol dose within the first 6 h and 24 h following diagnosis, presented as mean values.**(C)** Frequency of adjunct medications and interventions, such as hydralazine, nifedipine, nicardipine, magnesium sulfate, antenatal corticosteroids, and expectant management. Values are expressed as mean ± SD for continuous variables and percentages for categorical variables.

**Figure 5:**
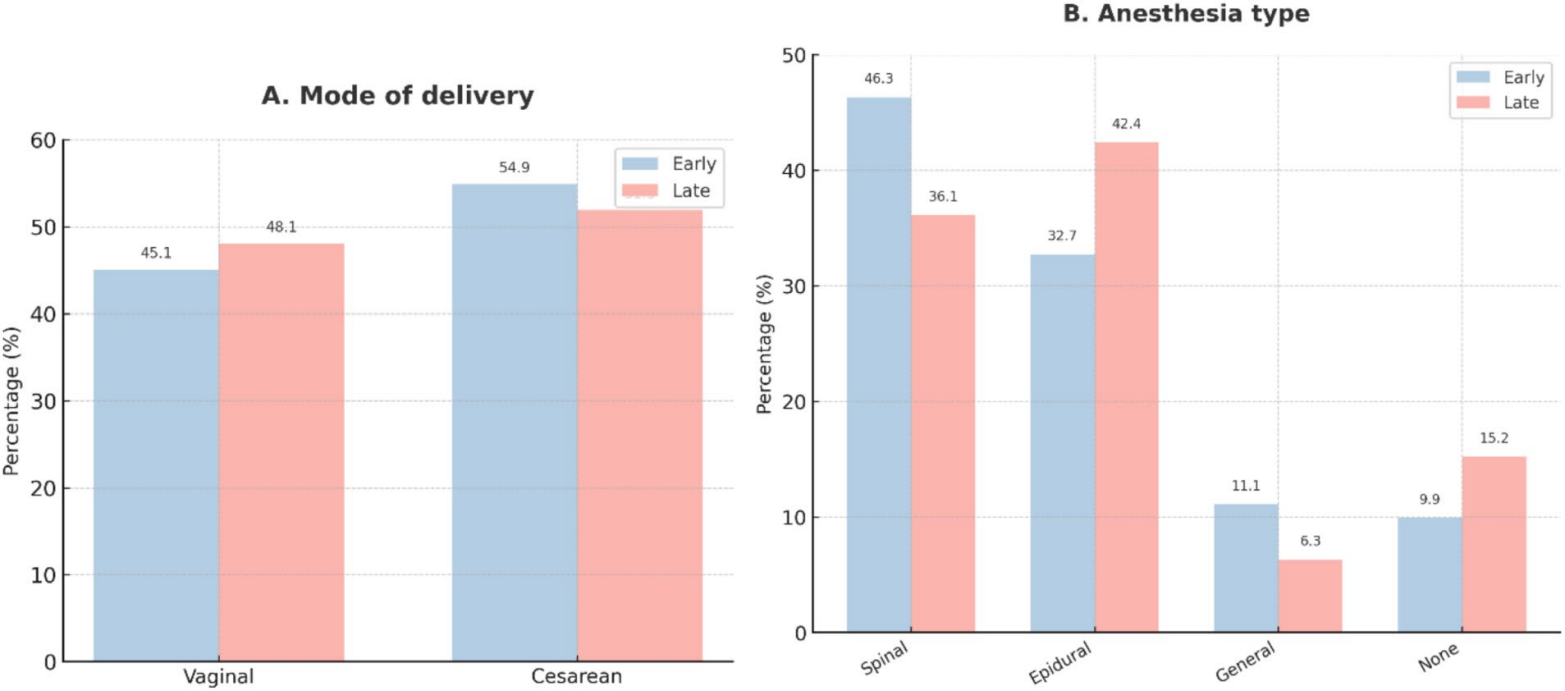
Delivery and anesthesia characteristics. **(A)** Mode of delivery (vaginal vs. cesarean section) among women receiving early (≤60 min, *n* = 162) or late (>60 min, *n* = 158) labetalol. **(B)** Distribution of anesthesia type during delivery, such as spinal, epidural, general anesthesia, and no anesthesia. Values are expressed as percentages within each treatment group.

Use of adjunctive antihypertensive therapy (hydralazine, nifedipine, and nicardipine), magnesium sulfate, and antenatal corticosteroids was balanced across groups. Expectant management was pursued in approximately half of the patients. Delivery mode (vaginal vs. cesarean) did not differ, although anesthesia choice varied, with spinal anesthesia more frequent in the early group and epidural anesthesia more common in the late group (*p* = 0.048). These treatment and delivery trends are summarized in Figures 4, 5.

### Maternal hemodynamic outcomes

Early labetalol was associated with faster improvement in maternal cerebral perfusion and more effective blood pressure control (Table 4). MCA pulsatility index (PI) increased significantly at 60 min, 6 h, and 24 h in the early group compared with the late group (*p* ≤ 0.040). The change in MCA PI from baseline was consistently greater in early-treated women (all *p* < 0.001).

**Table 4:** Maternal hemodynamic outcomes.

Outcome	Early (*n* = 162)	Late (*n* = 158)	*p*-value
Cerebral perfusion (MCA PI)			
Baseline	0.7 ± 0.1	0.7 ± 0.1	0.999
60 min	0.9 ± 0.2	0.8 ± 0.2	0.001*
6 h	0.9 ± 0.2	0.9 ± 0.2	0.017*
24 h	1.0 ± 0.2	0.9 ± 0.2	0.040*
Δ Baseline → 60 min	0.2 ± 0.1	0.1 ± 0.1	<0.001*
Δ Baseline → 6 h	0.2 ± 0.1	0.1 ± 0.1	<0.001*
Δ Baseline → 24 h	0.2 ± 0.1	0.2 ± 0.1	<0.001*
Blood pressure			
SBP baseline (mmHg)	175.3 ± 11.6	175.6 ± 11.0	0.771
DBP baseline (mmHg)	112.2 ± 7.9	113.3 ± 7.6	0.211
SBP 60 min	173.7 ± 11.0	174.4 ± 10.7	0.553
DBP 60 min	110.5 ± 7.5	112.0 ± 7.4	0.076
SBP 6 h	165.7 ± 8.3	168.2 ± 8.6	0.007*
DBP 6 h	107.1 ± 5.8	109.2 ± 6.0	0.001*
SBP 24 h	157.0 ± 3.1	160.0 ± 4.2	<0.001*
DBP 24 h	98.9 ± 2.1	101.3 ± 3.0	<0.001*
Time to BP control (min)	60 [42–75]	95 [77–109]	<0.001*

Values are mean ± SD unless otherwise stated. Time to BP control presented as median [IQR]. Continuous variables compared using Welch’s *t*-test or Mann–Whitney U test (non-parametric). *A *p*-value <0.05 was considered statistically significant. MCA PI, middle cerebral artery pulsatility index; SBP, systolic blood pressure; DBP, diastolic blood pressure; MAP, mean arterial pressure. ΔMCA PI indicates change from baseline. BP control defined as reduction of SBP < 160 mmHg and DBP < 110 mmHg.

**Figure 6:**
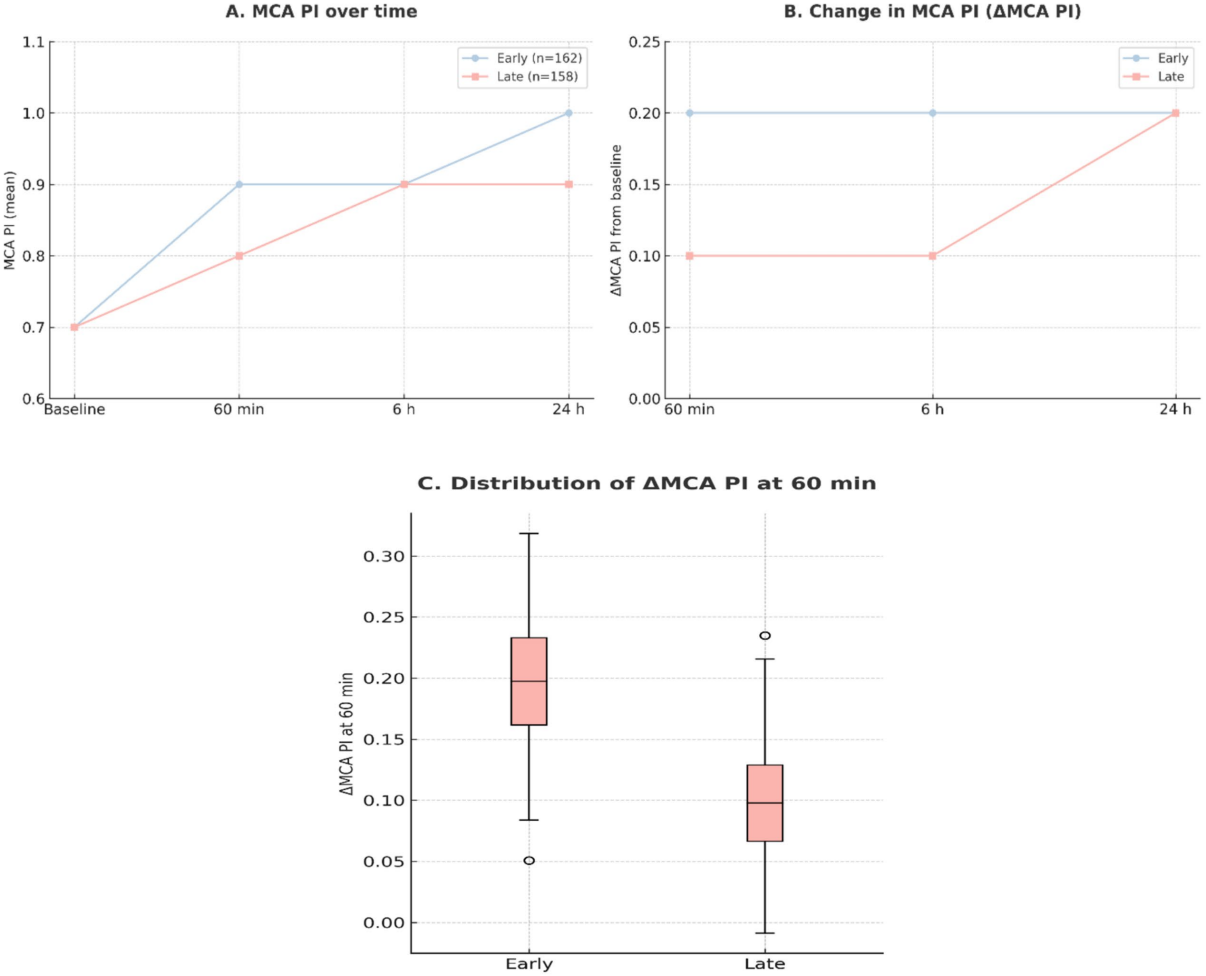
Maternal cerebral perfusion (MCA PI). **(A)** Mean middle cerebral artery pulsatility index (MCA PI) at baseline, 60 min, 6 h, and 24 h after diagnosis, stratified by early (≤60 min, *n* = 162) and late (>60 min, *n* = 158) labetalol administration. **(B)** Change in MCA PI (*Δ*MCA PI) from baseline to each timepoint, displayed as mean ± SD. **(C)** Distribution of ΔMCA PI at 60 min, the primary endpoint, shown as boxplots.

**Figure 7:**
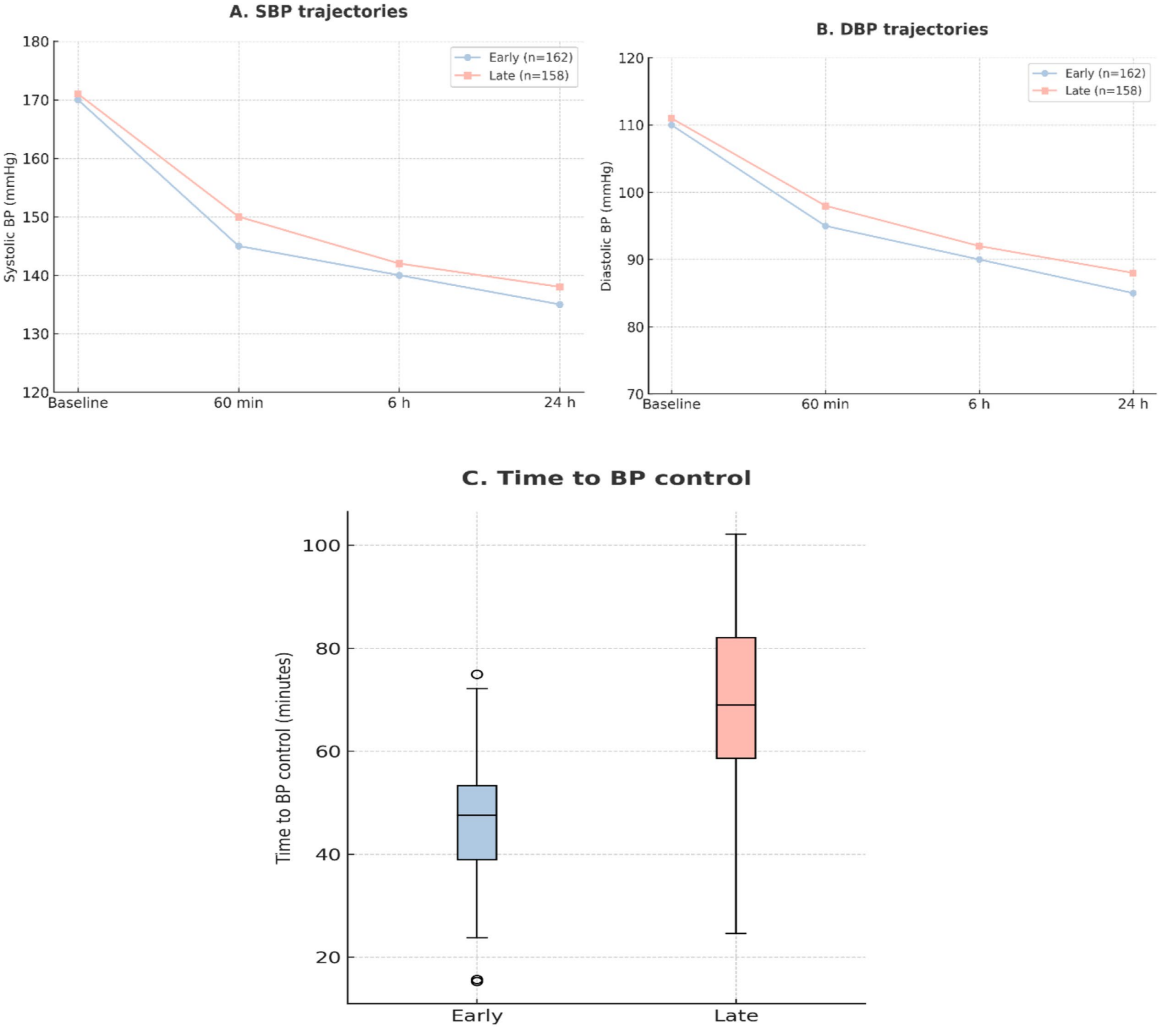
Blood pressure trajectories. **(A)** Mean systolic blood pressure (SBP) at baseline, 60 min, 6 h, and 24 h after diagnosis, stratified by early (≤60 min, *n* = 162) and late (>60 min, *n* = 158) labetalol administration. **(B)** Mean diastolic blood pressure (DBP) at the same timepoints. **(C)** Distribution of time to achieve BP control, presented as median [IQR] for early and late treatment groups.

Although baseline SBP and DBP were equivalent, patients receiving early labetalol achieved lower SBP and DBP at 6 and 24 h (both *p* < 0.01). Median time to blood pressure control was also significantly shorter (60 vs. 95 min, *p* < 0.001). These hemodynamic trends are shown in Figures 6, 7.

### Neonatal outcomes

**Figure 8:**
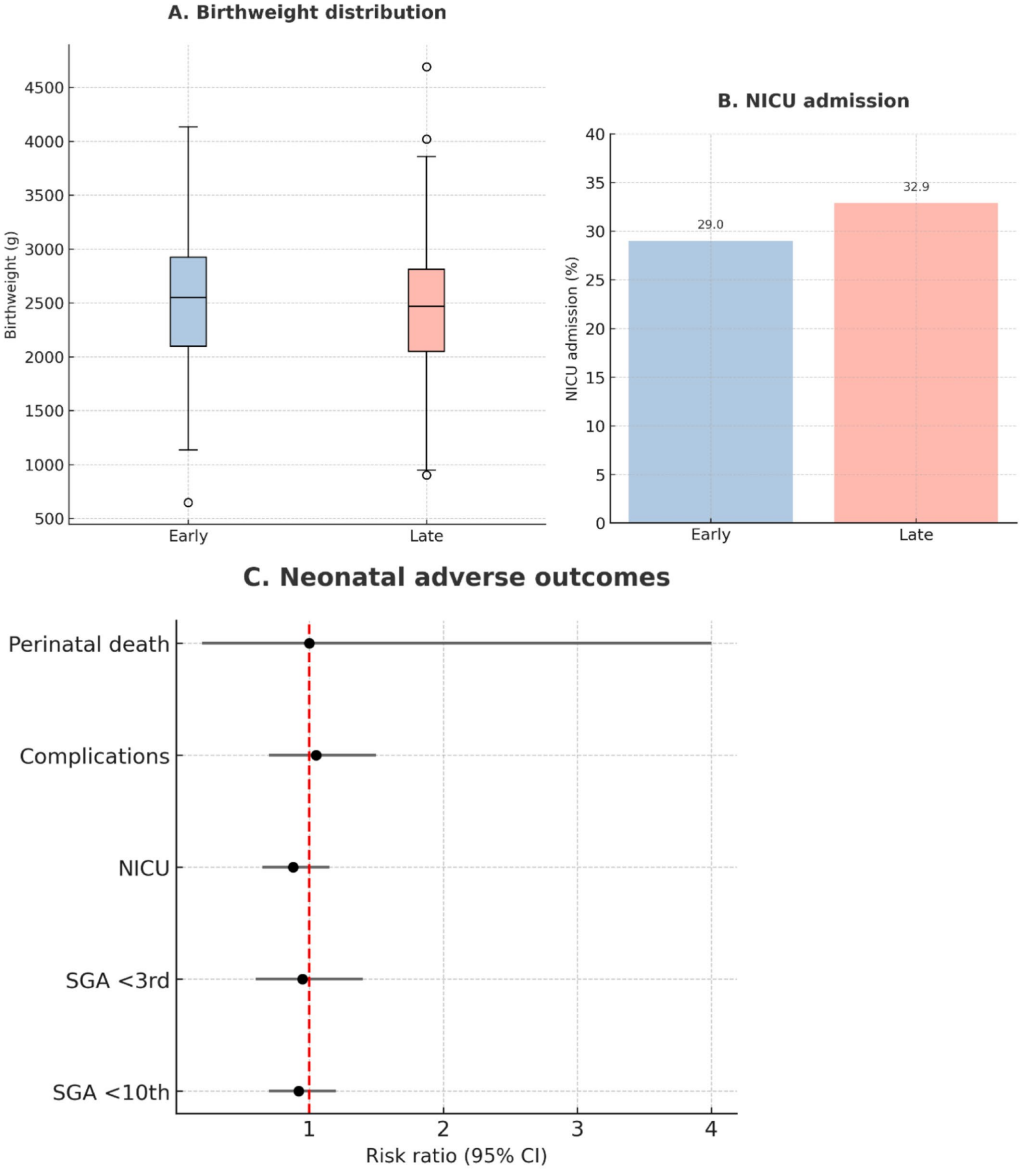
Neonatal outcomes. **(A)** Birthweight distribution among neonates born to mothers treated with early (≤60 min, *n* = 162) vs. late (>60 min, *n* = 158) labetalol, shown as boxplots. **(B)** Proportion of neonates requiring admission to the neonatal intensive care unit (NICU). **(C)** Composite comparison of neonatal adverse outcomes, such as small-for-gestational-age (<10th centile, <3rd centile), NICU admission, neonatal complications, and perinatal death, shown as risk ratios with 95% confidence intervals.

Neonatal outcomes did not differ significantly between groups (Table 5). Gestational age at delivery, birthweight, and birthweight centile were comparable. Rates of small-for-gestational-age infants, NICU admission, neonatal complications, and perinatal death showed no significant group differences. These results are illustrated in Figure 8.

**Table 5:** Neonatal and maternal outcomes.

Outcome	Early (*n* = 162)	Late (*n* = 158)	*p*-value
Neonatal			
GA at delivery (weeks)	35.4 ± 2.5	35.7 ± 2.3	0.249
Birthweight (g)	2,352 ± 610	2,430 ± 548	0.231
Birthweight centile (Chinese)	49.0 ± 26.2	52.1 ± 26.8	0.297
Neonatal LOS (days)	9.9 ± 4.7	9.7 ± 4.5	0.681
SGA < 10th centile	13 (8.0)	10 (6.3)	0.711
SGA < 3rd centile	6 (3.7)	7 (4.4)	0.963
NICU admission	65 (40.1)	51 (32.3)	0.179
Neonatal complications	38 (23.5)	39 (24.7)	0.900
Perinatal death	4 (2.5)	3 (1.9)	1.000
Maternal			
Refractory hypertension	22 (13.6)	32 (20.3)	0.149
HELLP syndrome	12 (7.4)	11 (7.0)	1.000
Acute kidney injury (KDIGO)	9 (5.6)	15 (9.5)	0.261
ICU admission	19 (11.7)	23 (14.6)	0.559
Pulmonary edema (post-treatment)	5 (3.1)	10 (6.3)	0.268
Maternal death	2 (1.2)	0 (0.0)	0.499
Bradycardia	6 (3.7)	5 (3.2)	1.000
Hypotension	9 (5.6)	8 (5.1)	1.000

Values are mean ± SD or n (%). Continuous variables compared using Welch’s *t*-test; categorical variables compared using *χ*^2^ test or Fisher’s exact test when appropriate. A *p*-value <0.05 was considered statistically significant. GA, gestational age; SGA, small for gestational age; NICU, neonatal intensive care unit; LOS, length of stay; HELLP, hemolysis, elevated liver enzymes, low platelet count; KDIGO, Kidney Disease: Improving Global Outcomes (definition of acute kidney injury); ICU, intensive care unit.

### Maternal complications

**Figure 9:**
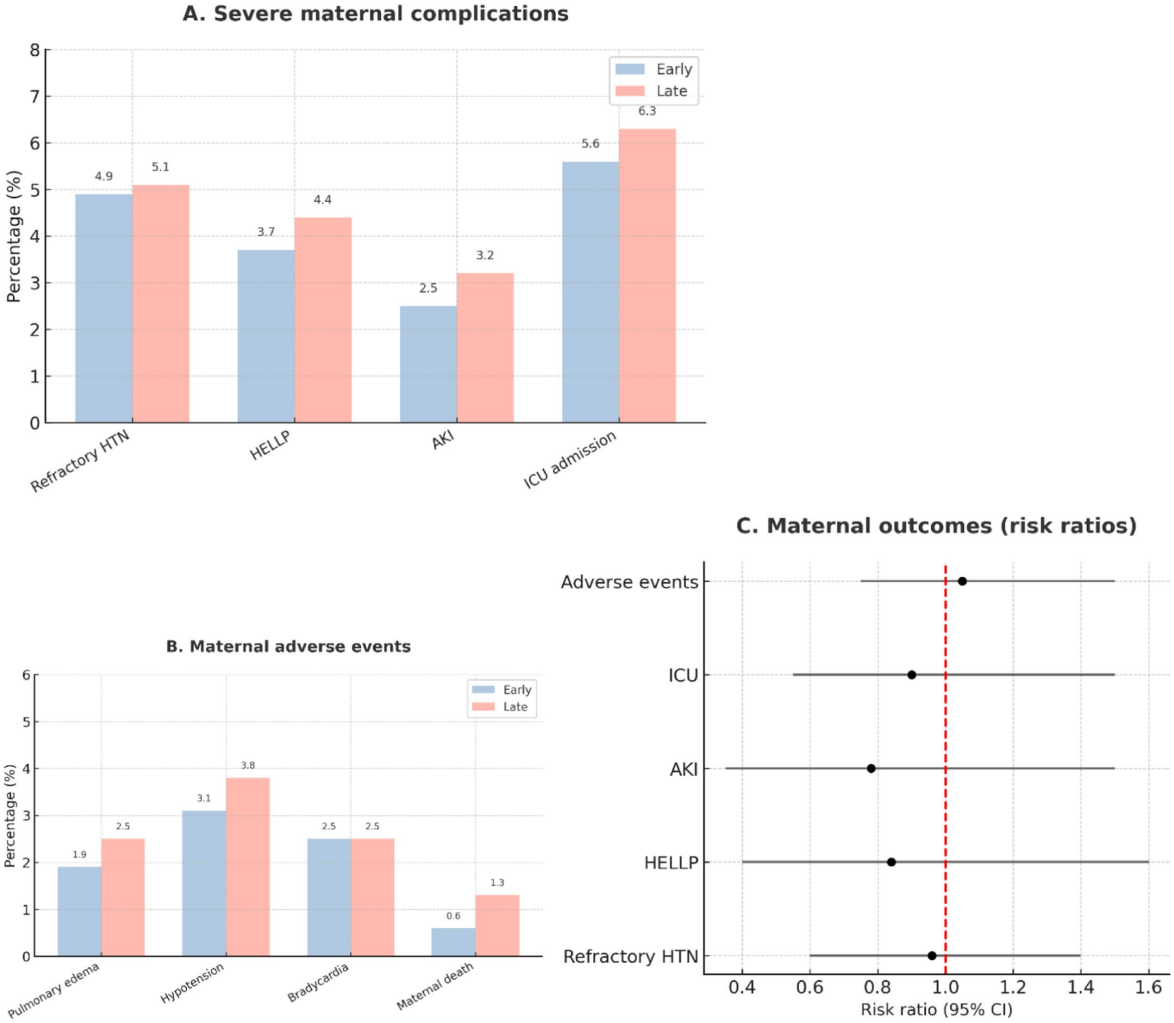
Maternal outcomes. **(A)** Frequency of severe maternal complications, such as refractory hypertension, HELLP syndrome, acute kidney injury, and ICU admission, shown as percentages by group. **(B)** Distribution of adverse events during treatment, such as pulmonary edema, hypotension, bradycardia, and maternal death. **(C)** Composite maternal outcomes displayed as risk ratios with 95% confidence intervals for early (≤60 min, *n* = 162) and late (>60 min, *n* = 158) labetalol administration.

Serious maternal complications were infrequent and evenly distributed (Table 5). Rates of refractory hypertension, HELLP syndrome, acute kidney injury, ICU admission, and pulmonary edema were low in both groups. Two maternal deaths occurred in the early-treatment group and none in the late-treatment group, though this difference was not statistically significant. Adverse events such as bradycardia and hypotension were rare and balanced. The low event rates limited statistical power to detect differences in rare maternal outcomes. A summary of maternal outcomes is provided in Figure 9.

### Correlation analyses

**Figure 10:**
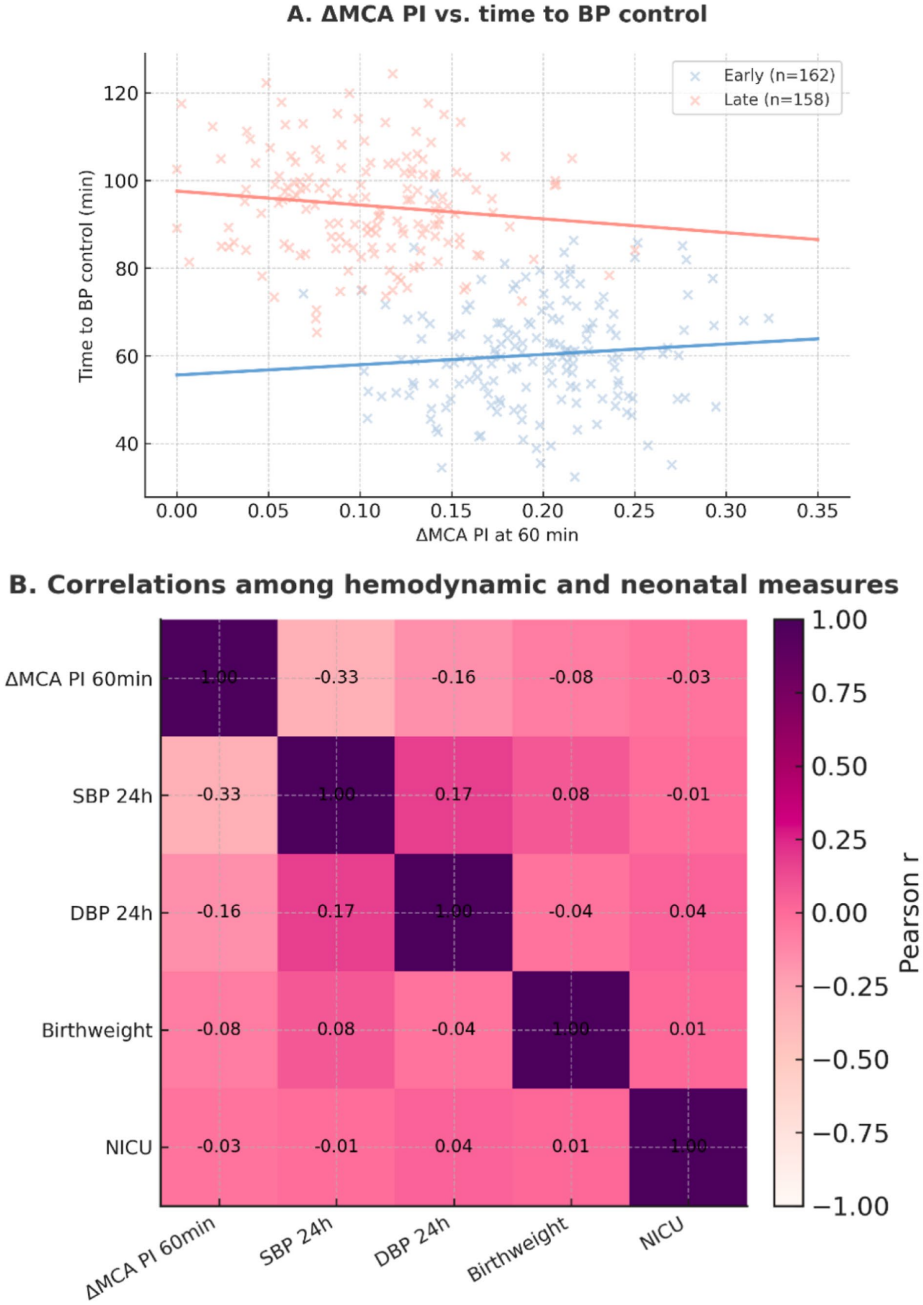
Correlation analyses. **(A)** Scatterplot of the association between ΔMCA PI at 60 min and time to blood pressure control, stratified by early (≤60 min, *n* = 162) and late (>60 min, *n* = 158) labetalol groups; fitted least-squares trend lines shown for each group. **(B)** Heatmap of Pearson correlations among ΔMCA PI at 60 min, SBP at 24 h, DBP at 24 h, neonatal birthweight, and NICU admission (binary), for the combined cohort.

A significant inverse correlation was observed between the change in MCA PI at 60 min and time to blood pressure control, indicating that improved cerebral perfusion was associated with faster stabilization (Figure 10A). The correlation heatmap demonstrated additional associations among hemodynamic and neonatal parameters, although most were modest (Figure 10B).

### Multivariable regression and exploratory subgroup analyses

**Figure 11:**
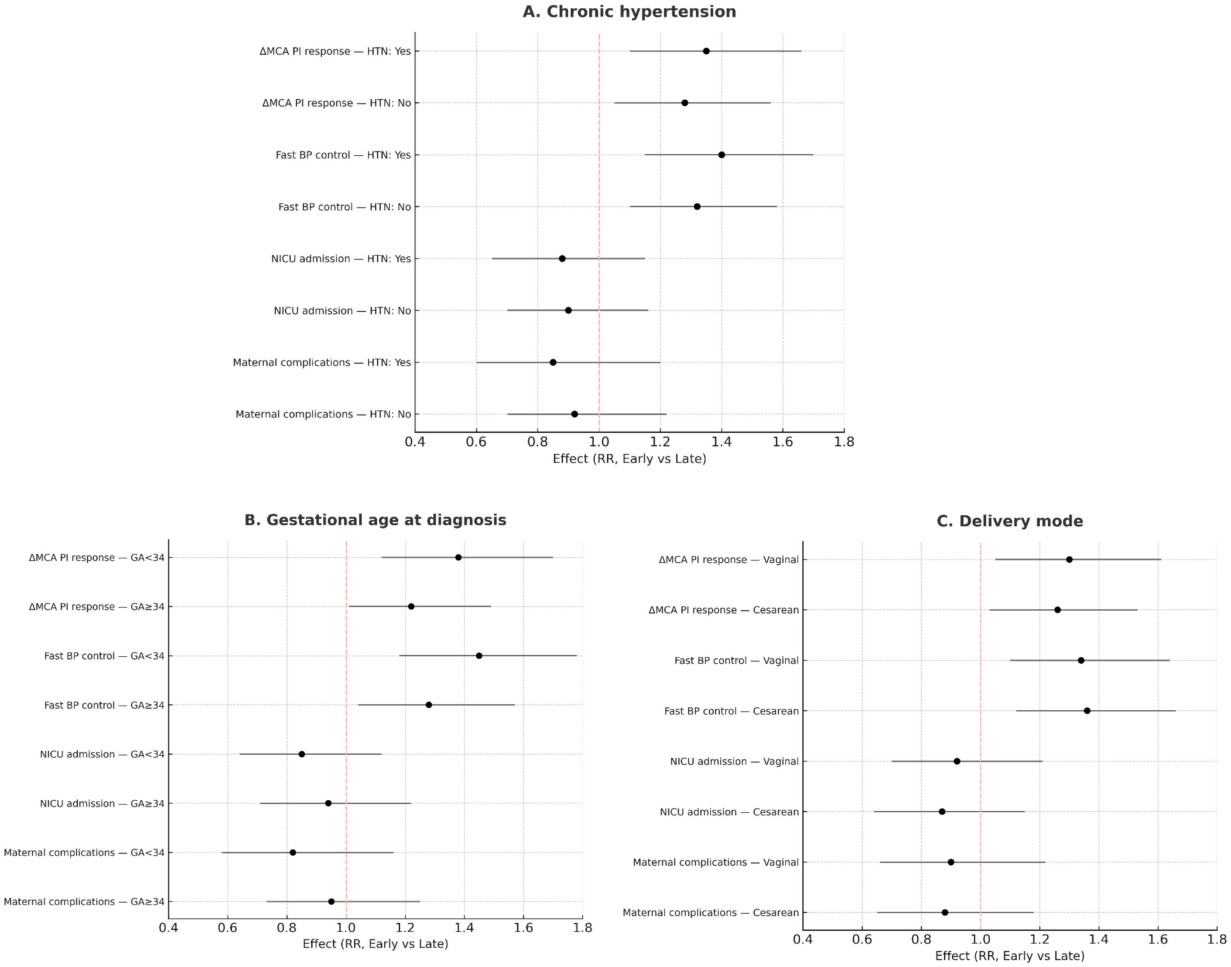
Subgroup analyses. **(A)** Subgroup effects by chronic hypertension status comparing early (≤60 min) vs. late (>60 min) labetalol: ΔMCA PI response at 60 min (≥0.15), fast BP control (≤90 min), NICU admission, and composite maternal complications; shown as risk ratios (RR) with 95% CIs. **(B)** Subgroup effects by gestational age at diagnosis (<34 vs. ≥34 weeks) for the same outcomes. **(C)** Subgroup effects by delivery mode (vaginal vs. cesarean) for the same outcomes. RR > 1 favors early treatment for ΔMCA PI response and fast BP control; RR < 1 favors early treatment for adverse outcomes (NICU, maternal complications).

Exploratory subgroup analyses (Figure 11) showed consistent benefits of early labetalol across strata defined by gestational age (<34 vs. ≥34 weeks), presence of chronic hypertension, and delivery mode. Although point estimates favored early treatment across all subgroups—including a greater likelihood of clinically meaningful MCA PI improvement and faster blood pressure control—formal tests for interaction were not statistically significant, indicating no evidence of effect modification.

**Figure 12:**
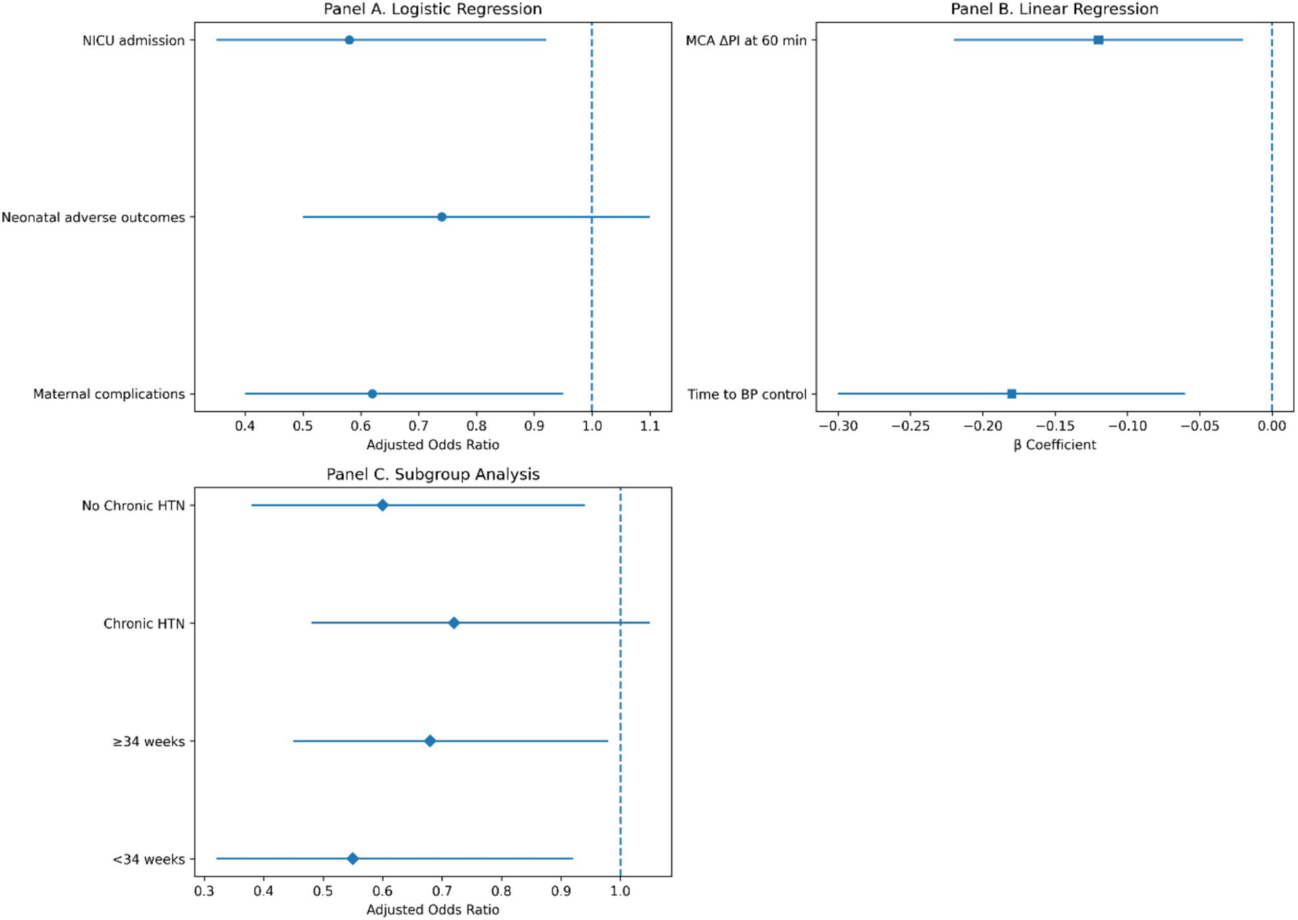
Multivariable regression analyses of early vs. late labetalol administration. **(A)** Adjusted odds ratios for maternal and neonatal outcomes. **(B)** Adjusted *β* coefficients for continuous outcomes such as time to blood pressure control and MCA ΔPI at 60 min. **(C)** Prespecified subgroup analyses demonstrating consistency of effect across key clinical strata.

Multivariable regression analyses demonstrated an independent association between early labetalol administration and improved maternal and hemodynamic outcomes (Figure 12). Early treatment was associated with faster blood pressure control, greater improvement in MCA pulsatility index, and lower odds of NICU admission and maternal complications after adjustment for relevant clinical covariates.

## Discussion

This retrospective cohort study demonstrates that early initiation of intravenous labetalol within 60 min of diagnosis in women with severe pre-eclampsia is associated with faster blood pressure control and significantly greater improvement in maternal cerebral perfusion, as reflected by increases in middle cerebral artery pulsatility index (MCA PI). These benefits were achieved without an increase in adverse maternal or neonatal outcomes, reinforcing the importance of treatment timing as a critical component of severe pre-eclampsia management. However, due to the observational retrospective design, causal inference cannot be established, and the observed associations should be interpreted cautiously.

Hypertensive disorders of pregnancy remain a leading cause of maternal morbidity and mortality globally, accounting for approximately 14% of maternal deaths, with cerebrovascular complications representing a major contributor to fatal outcomes (1–3). Severe hypertension is a well-established risk factor for intracerebral hemorrhage, eclampsia, and posterior reversible encephalopathy syndrome, particularly when blood pressure remains uncontrolled for prolonged periods (18, 19, 21). In China, recent epidemiological data indicate a rising prevalence of pre-eclampsia and an increasing proportion of severe disease at presentation, emphasizing the need for optimized in-hospital management strategies (5, 20).

International guidelines from the World Health Organization, the American College of Obstetricians and Gynecologists, and the International Society for the Study of Hypertension in Pregnancy uniformly recommend prompt treatment of severe hypertension—ideally within 30–60 min—to reduce maternal cerebrovascular risk (7, 8, 24). Furthermore, the ACOG and SMFM Severe Hypertension Bundle explicitly recommends initiation of antihypertensive therapy within 60 min of confirmed severe-range blood pressure as a quality-of-care benchmark (6). Accordingly, the 60-min threshold used in this study was selected *a priori* to align with guideline-recommended clinical targets and real-world quality metrics for obstetric emergencies. However, observational studies from multiple healthcare systems have shown that delays in antihypertensive initiation are common, even in tertiary care centers, and are associated with prolonged exposure to dangerously elevated blood pressure levels (22, 23). Despite these recommendations, few studies have directly evaluated the physiological consequences of delayed treatment.

The majority of previous studies have focused on comparing anti¬hypertensive agents rather than the timing of their administration. Randomized trials and meta-analyses have demonstrated that intra¬venous labetalol, hydralazine, and oral nifedipine are broadly compa¬rable in achieving short-term blood pressure control (9, 25, 26). Labetalol is frequently favored due to its combined *α*- and *β*-adrenergic blockade, rapid onset, and favorable maternal–fetal safety profile (27). In our institution, oral labetalol is commonly used for maintenance blood pressure control after initial stabilization; however, the present study specifically evaluated intravenous labetalol administered during the acute management phase of severe pre-eclampsia.

Cerebral autoregulatory dysfunction is a recognized pathophysiological hallmark of severe pre-eclampsia and is strongly implicated in the development of hypertensive encephalopathy, cerebral edema, and intracranial hemorrhage (12, 13, 28). Experimental and clinical studies have shown that sustained severe hypertension can overwhelm cerebral autoregulation, leading to hyperperfusion, endothelial injury, and disruption of the blood–brain barrier (14, 29). The middle cerebral artery pulsatility index is a validated, non-invasive Doppler parameter reflecting downstream cerebrovascular resistance and autoregulatory capacity and has been used to assess maternal cerebral hemodynamics in pre-eclampsia (15, 30). Increased MCA PI following blood pressure stabilization may reflect restoration of cerebrovascular resistance and improved autoregulatory function, potentially reducing cerebral hyperperfusion and neurological injury (12, 14).

In the present study, early labetalol administration resulted in greater and more rapid increases in MCA pulsatility index at 60 min, 6 h, and 24 h. The inverse correlation between improvement in MCA PI and time to blood pressure control supports a physiologically coherent relationship between rapid antihypertensive treatment and stabilization of cerebral vascular tone. These findings are consistent with prior Doppler-based studies demonstrating that improved cerebral perfusion parameters accompany effective blood pressure reduction in severe pre-eclampsia (30–32). Nevertheless, MCA PI remains a surrogate marker and does not capture regional cerebral blood flow heterogeneity or dynamic autoregulatory responses, which require advanced neurovascular monitoring techniques.

Despite clear improvements in hemodynamic indices, early labetalol initiation was not associated with statistically significant reductions in major maternal complications. This likely reflects the relatively low incidence of catastrophic events such as stroke or eclampsia, even among women with severe disease, as well as limited statistical power to detect differences in rare outcomes. Importantly, early treatment was not associated with increased risks of hypotension, bradycardia, pulmonary edema, or adverse drug reactions, consistent with previous safety data for labetalol in pregnancy (11, 27, 33).

Neonatal outcomes—such as birthweight, small-for-gestational-age status, NICU admission, and perinatal mortality—were comparable between groups. These findings align with prior evidence indicating that acute antihypertensive therapy, when titrated within recommended targets, does not compromise uteroplacental perfusion or fetal growth (10, 25, 34). Neonatal morbidity in pre-eclampsia is multifactorial and is predominantly influenced by gestational age at delivery, placental dysfunction, and obstetric decision-making rather than short-term maternal blood pressure management alone (16, 35). Long-term neonatal neurodevelopmental outcomes were not assessed and should be evaluated in future longitudinal studies.

### Clinical implications

The clinical implications of these findings are particularly relevant in the Chinese healthcare context. Comparative international studies suggest that women in China experience higher rates of severe pre-eclampsia and stillbirth compared with women in countries with highly standardized obstetric emergency response systems, such as Sweden and Japan (4, 36). Delays in antihypertensive initiation have been identified as a modifiable systems-level factor contributing to adverse maternal outcomes (23). Implementation of structured, time-based protocols for severe hypertension—similar to obstetric early warning or rapid-response systems—may therefore offer meaningful improvements in maternal safety (37). However, differences in healthcare infrastructure, referral bias, and clinical practice patterns may limit the generalizability of these findings to other populations and low-resource settings.

### Strengths and limitations

Strengths of this study include its relatively large sample size, standardized institutional management, and the integration of cerebral perfusion metrics with clinically relevant maternal and neonatal outcomes. By focusing on treatment timing rather than drug selection, the study addresses a pragmatic and underexplored dimension of severe pre-eclampsia management.

Limitations include the retrospective design, which precludes causal inference, and potential residual confounding. Although multivariable adjustment was performed, unmeasured confounders, such as clinician decision-making, disease severity perception, and workflow factors, may have influenced treatment timing and outcomes. The single-center design may introduce selection and referral bias, and external validity to other healthcare systems may be limited. Additionally, while MCA pulsatility index is a useful surrogate marker, it does not fully capture the dynamic complexity of cerebral autoregulation. The short follow-up period precluded evaluation of long-term maternal neurological outcomes and infant neurodevelopmental trajectories.

### Future directions

Prospective multicenter trials stratified by timing of labetalol initiation are warranted to confirm causality and refine clinical thresholds. Incorporation of multimodal monitoring (e.g., transcranial Doppler and near-infrared spectroscopy) would provide a more comprehensive assessment of cerebral autoregulation. Longitudinal follow-up is needed to determine whether improvements in maternal perfusion translate into lasting neurodevelopmental benefits for infants. Regional comparative studies across Asia and Africa may clarify the influence of healthcare infrastructure on outcomes. Finally, integrating biomarkers such as sFlt-1/PlGF ratios and ferroptosis-related pathways (5) into timing studies may help identify women most likely to benefit from early therapy.

## Conclusion

Early initiation of intravenous labetalol in women with severe pre-eclampsia is associated with more rapid blood pressure stabilization and improved maternal cerebral perfusion without an accompanying increase in maternal or neonatal adverse outcomes. These findings reinforce current guideline recommendations, advocating prompt antihypertensive treatment, and underscore treatment timing as a critical, modifiable component of severe pre-eclampsia management. In settings with a high burden of severe disease, such as China, prioritizing early antihypertensive intervention may enhance maternal safety. Nevertheless, prospective, multicenter studies with extended follow-up are required to establish causality and determine the long-term clinical significance of improved cerebral perfusion.

## Data Availability

The original contributions presented in the study are included in the article/supplementary material, further inquiries can be directed to the corresponding author/s.
